# Correction to “Death‐associated protein kinase 1 suppresses hepatocellular carcinoma cell migration and invasion by upregulation of DEAD‐box Helicase 20”

**DOI:** 10.1111/cas.70333

**Published:** 2026-02-08

Huang Y, Wang C, Li K, et al. Death‐associated protein kinase 1 suppresses hepatocellular carcinoma cell migration and invasion by upregulation of DEAD‐box helicase 20. *Cancer Sci*. 2020;111:2803–2813. https://doi.org/10.1111/cas.14499

There were errors in image usage, incorrect annotations and upload errors in the above article. We have made corrections. Most importantly, these corrections do not affect the validity of the conclusions.

The corrected Figure 1, 2, 3, S1 and S2 are as follows:

Figure 1

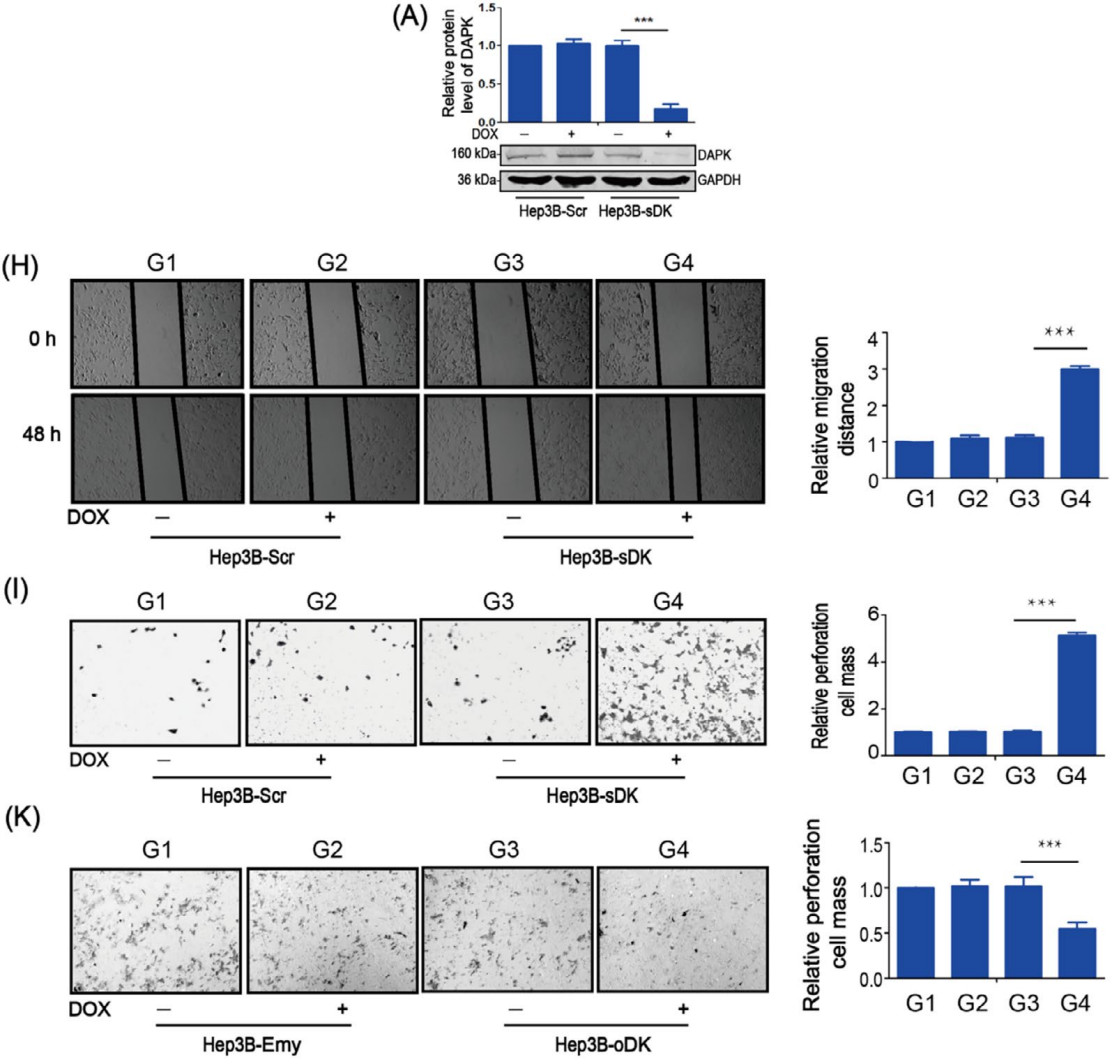

Figure 2

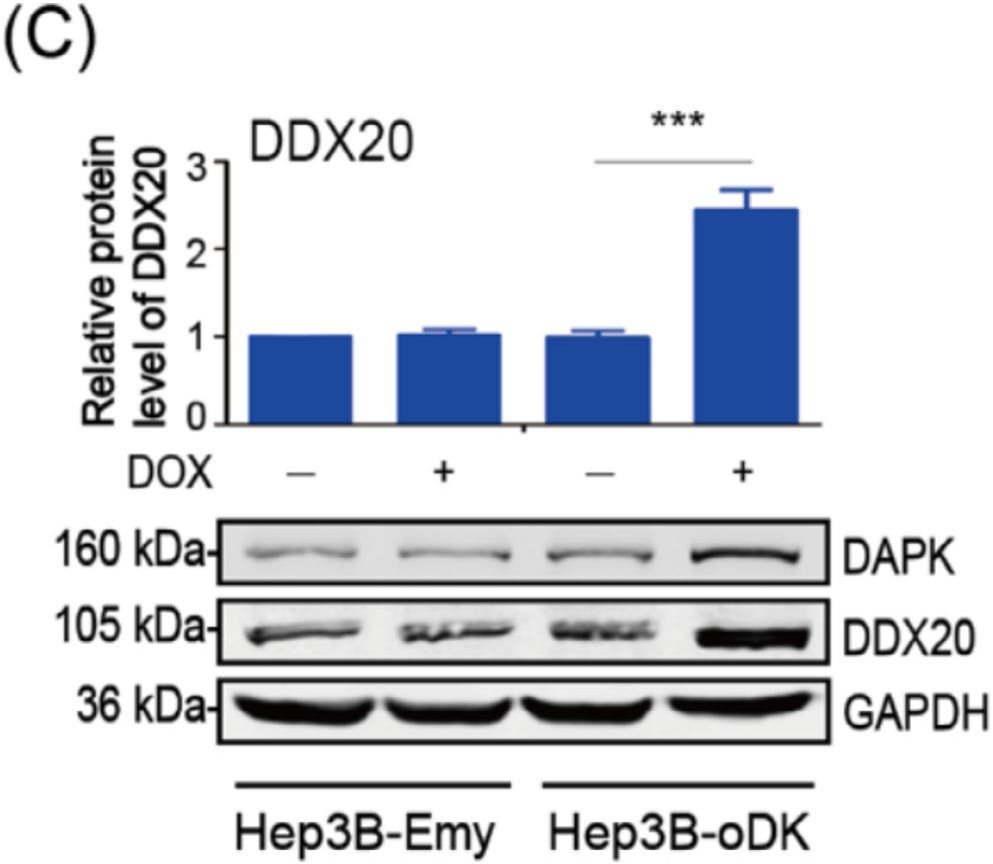

Figure 3

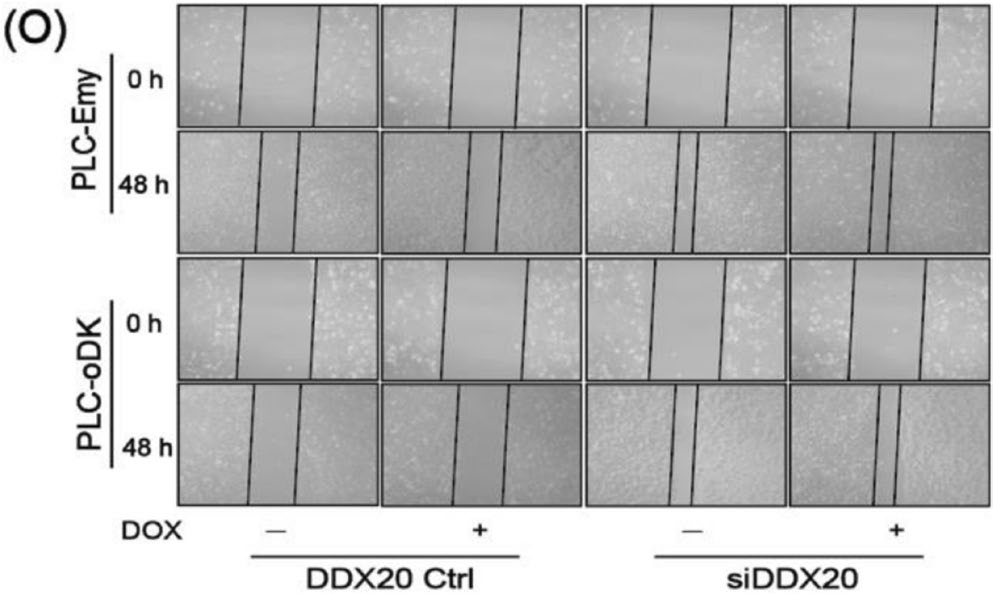

Figure S1

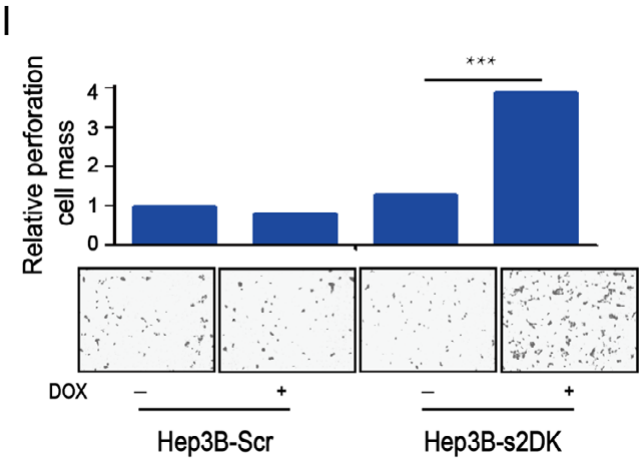

Figure S2

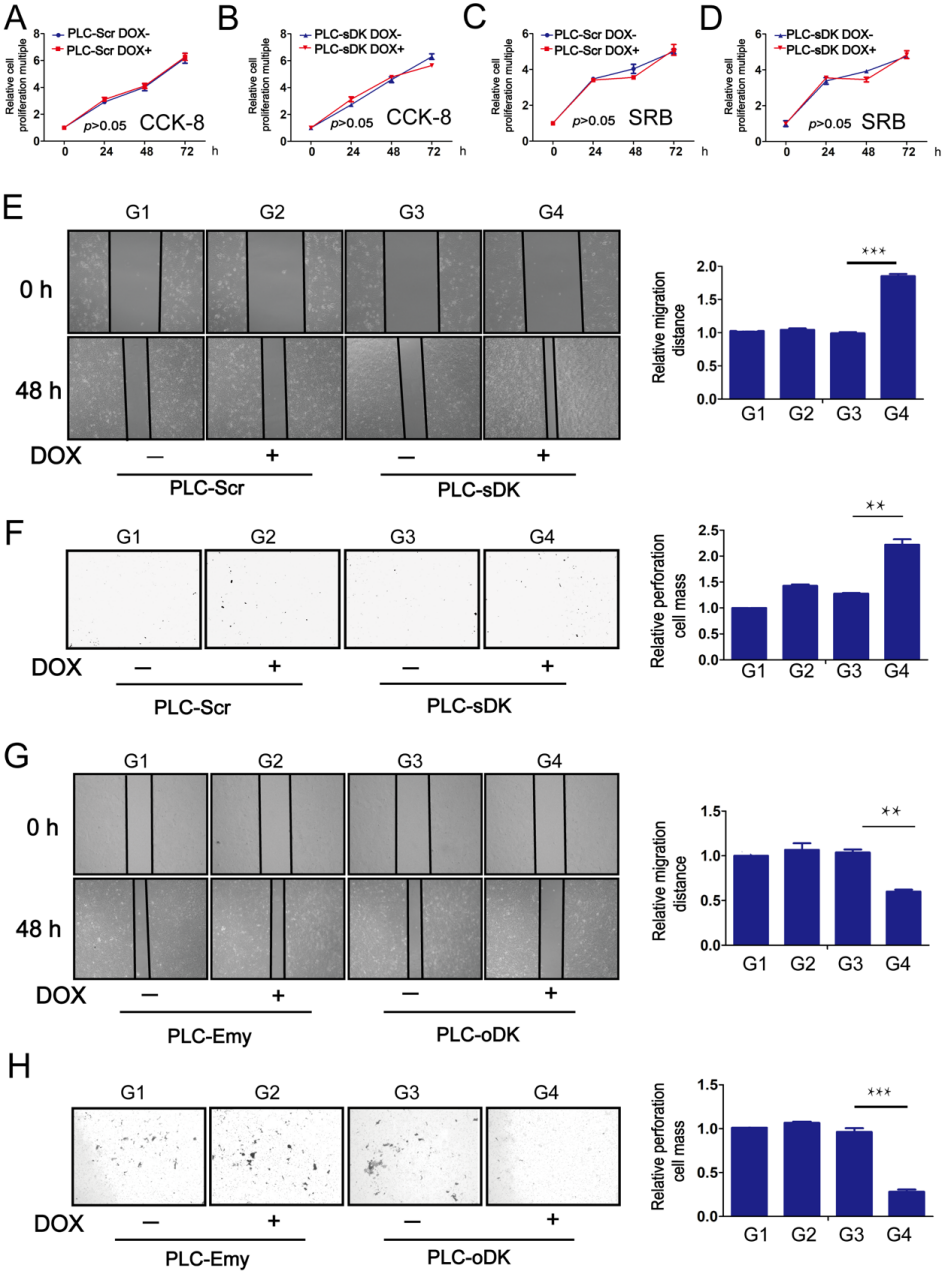

We apologize for these errors.

